# Frequency Analysis of Atrial Fibrillation From the Surface Electrocardiogram

**Published:** 2004-07-01

**Authors:** Daniela Husser, Martin Stridh, Leif Sornmo, S. Bertil Olsson, Andreas Bollmann

**Affiliations:** * Department of Cardiology, Good Samaritan Hospital, Los Angeles, USA; † Harbor-UCLA Medical Center, Los Angeles, USA; ‡ Departments of Electroscience, Lund University, Lund, Sweden; § Departments of Cardiology, Lund University, Lund, Sweden

**Keywords:** atrial fibrillation, ECG signal processing, electrical remodeling, antiarrhythmic drug monitoring, cardioversion

## Abstract

Atrial fibrillation (AF) is the most common arrhythmia encountered in clinical practice. Neither the natural history of AF nor its response to therapy are sufficiently predictable by clinical and echocardiographic parameters.

Atrial fibrillatory frequency (or rate) can reliably be assessed from the surface electrocardiogram (ECG) using digital signal processing (filtering, subtraction of averaged QRST complexes, and power spectral analysis) and shows large inter-individual variability. This measurement correlates well with intraatrial cycle length, a parameter which appears to have primary importance in AF domestication and response to therapy. AF with a low fibrillatory rate is more likely to terminate spontaneously, and responds better to antiarrhythmic drugs or cardioversion while high rate AF is more often persistent and refractory to therapy.

In conclusion, frequency analysis of AF seems to be useful for non-invasive assessment of electrical remodeling in AF and may subsequently be helpful for guiding AF therapy.

In recent years, mechanisms leading to atrial fibrillation (AF) induction and maintenance have begun to be explored and rapidly evolving therapies including new antiarrhythmic drugs, antitachycardia pacing algorithms and catheter ablation techniques have been introduced in clinical practice. Nevertheless, outcome of AF patients is still unsatisfactory which - to the greatest extent - can be attributed to the progressing nature of this arrhythmia and high AF recurrence rates following restoration of sinus rhythm. Moreover, AF treatment (e.g. rate vs. rhythm control, choice of antiarrhythmic drugs or device therapy, curative ablation) may be viewed as being based on trial and error, since no test is able to predict the natural history of this arrhythmia or its response to treatment. Subsequently, current AF management guidelines [1] provide no treatment recommendations that “take the various mechanisms and patterns of AF into account”. Thus, it seems desirable to develop tests that quantify AF disease state and guide AF management [2].

Virtually in every patient with AF, a standard surface electrocardiogram (ECG) is acquired, the main purposes being confirmation of arrhythmia presence and determination of ventricular rate. Fibrillation waves have, moreover, various appearances ranging from fine to coarse and from disorganized to organized, flutter-like. Interestingly, the mechanisms behind the various appearances of the fibrillatory process on the ECG and the possible prognostic information contained within fibrillation waves have just very recently begun to be explored.

The aim of this article is twofold; (1) to describe novel frequency analysis techniques of the surface ECG for characterization of the fibrillatory process, and (2) to present possible applications of these methods, namely for (a) exploring autonomic modulation of AF, (b) monitoring and predicting antiarrhythmic drug effects, and (c) predicting AF recurrence following restoration of sinus rhythm. For this purpose, Medline-listed, peer-reviewed studies are included in this review.

## Rationale for Using Atrial Fibrillatory Rate to Characterize Human AF

Shortening of atrial refractoriness and maladaptaion to rate are hallmarks of atrial electrical remodeling in AF [3].. During AF, re-excitation occurs during the repolarization phase of the preceding electrical wave, implying that local excitation almost always occurs without any obvious latency beyond the refractory period. Subsequently, the average atrial fibrillatory rate is likely to reflect averaged refractoriness at any part of the tissue involved. This assumption has been independently verified in both animal [4]. and human AF [5,6]. Hence, the length of the averaged atrial fibrillatory cycle (which is inversely related to fibrillatory frequency or rate) can be used as an index of the averaged atrial myocardial refractoriness and subsequently AF organization [7]. The importance of shortened refractoriness and subsequently its transformation into short atrial cycles or high rates as assessed by direct intraatrial measurements for AF progression and response to therapy has been well established. Results will be reported as cycle length in milliseconds (ms) if they were obtained from intraatrial recordings. If they were obtained from surface recordings, they will be reported as fibrillatory rate in fibrillations per minute (fpm, the rationale to use this expression is discussed in depth below).

Firstly, spontaneous arrhythmia behavior is related to baseline fibrillatory cycle length. Asano et al induced AF with rapid pacing in 30 patients undergoing electrophysiologic study [8]. Patients in whom AF terminated spontaneously (n=20) had an average fibrillatory cycle lenghth of 176 ms (341 fpm), significantly lower than the 157 ms (382 fpm) recorded in the group of patients where the arrhythmia persisted (n=10). Similar observations were made by Boahene et al who also measured the fibrillatory cycle length from the right atrium in 55 patients with Wolff-Parkinson-White syndrome [9]. These investigators also found that patients with sustained AF (n=45) had shorter mean cycle lengths compared to patients with non-sustained AF (n=10).

Secondly, response to antiarrhythmic drug therapy has been shown to be associated with baseline cycle length and drug-induced cycle length changes. Stambler and colleagues identified a mean right atrial cycle length of 160 ms (375 fpm) as a valuable cutoff-point for conversion to sinus rhythm with ibutilide [10]. No patient with shorter cycle length (higher rate) was converted by ibutilide, whereas conversion occured in 64 % of those patients with longer cycle length (lower rate). Similarly, Fujiki et al [11] by using spectral analysis of right atrial electrograms reported 100 % AF conversions with cibenzoline or procainamide if the baseline atrial cycle length was > 168 ms (< 357 fpm) as opposed to only 17 % in patients with shorter cycle lengths (higher rates). Moreover, AF conversion was associated with cycle length prolongation. AF terminated after class I drug administration in 88 %, if the atrial cycle length had been prolonged to > 210 ms (< 285 fpm), in contrast to only 10 % if the post-drug cycle length was shorter.

Finally, there exists a close relation between the degree of electrical remodeling expressed as refractoriness changes and AF recurrence following cardioversion. Manios et al found that patients who failed to shorten the monophasic action potential duration to less then 195 ms at a pacing cycle length of 350 ms were more likely to have AF recurrence [12]. This finding was explained by a more abnormal rate adaptation curve, which is in agreement to an early study performed by Attuel and colleagues showing that poor or absent rate adaptation of atrial refractory periods (that is, the normally close correlation between stimulation cycle length and refractory period was lost) is related with vulnerability to AF [13]. These findings have more recently been replicated in patients undergoing internal cardioversion [14]. In their study, Biffi et al identified both a shorter atrial effective refractoriness and abnormal rate adaptation as independent predictors for AF relapses, the latter being the strongest predictor.

Prompted by the likely usefulness of non-invasive test that assesses the average fibrillatory rate from the surface ECG for exploration of AF pathophysiology and AF managment, frequency analysis techniques were independently introduced by our groups [15,16], while two earlier studies proposed to use this technique to subdivide AF and atrial flutter [17] or automatically identify AF among different rhythms [18].

## Frequency Analysis Techniques

### Methodological Considerations

Using digital recording techniques, traditional ECG signals are recorded. In most studies atrial fibrillatory rate has been obtained by spectral analysis techniques of resting ECG recordings such as standard 12-lead [15,16] or (modified) orthogonal recordings [19-21]. The method has, however, also been applied to ambulatory ECG recordings using conventional ambulatory leads [22-24].

The accuracy of frequency analysis techniques strongly relies on the fact that the largest possible fibrillatory waves should be present for further signal processing. Therefore, it was suggested to analyze lead V1 when using a standard ECG or, as proposed previously by Waktare et al [19-25], a “bipolar modification of V1” covering the atria (low C1) when applying different ECG recording systems (e.g. Holter ECG) [19-20].

**Figure 1:**
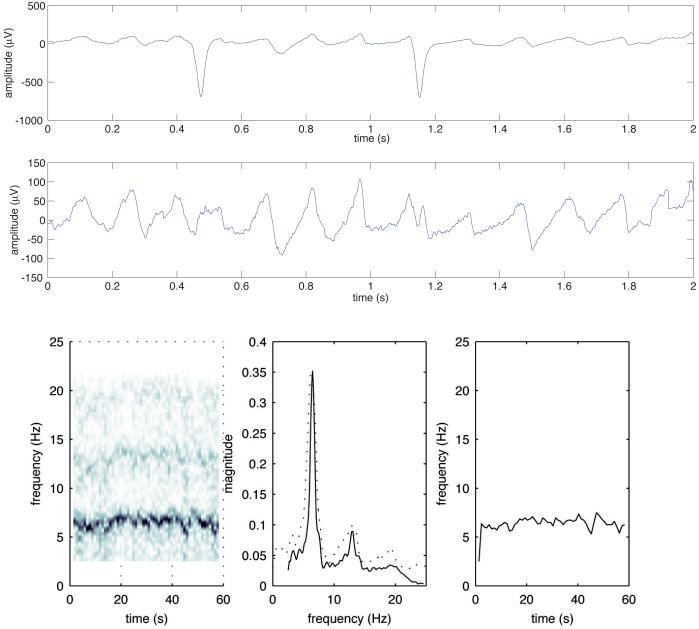
Frequency analysis of AF. Two seconds (out of a 60 second recording) of an ECG signal from a patient with AF (upper panel), and the same interval after QRST cancellation (middle panel, amplitude scale is magnified five times). This fibrillatory signal is then subjected to Fourier analysis. Time-frequency distribution (left box), power frequency spectrum in which the dominant fibrillatory rate is determined (middle box), frequency trend (right box).

Since the atrial and ventricular activities overlap spectrally, linear filtering techniques are not suitable for extraction of the fibrillatory signal from the surface ECG. Instead, subtraction of averaged QRST complexes needs to be performed producing a remaining atrial fibrillatory signal for further analysis (Figure 1). Originally, a fixed averaged QRST-complex was used for cancellation in individual leads. Further development of the method allowed an improved cancellation of the QRST-complex by taking changes in QRS morphology due to alterations in the electrical axis of the heart into account (spatiotemporal QRST cancellation) [26].

Following QRST cancellation, a power spectrum is obtained by using a windowing technique and Fourier analysis to process the remainder ECG. This as well as the associated windowing technique (window type, length and overlap) determine the appearance of the frequency power spectrum [15]. Variants of Fourier transform based methods including conventional Fourier analysis and spectral averaging techniques based on short overlapping segments have been applied to ECG segments ranging from 10 seconds to 5 minutes [15,16,27-29]. Typically a distinct spectral peak is obtained which corresponds to the most dominant fibrillatory rate of nearby endocardial sites [15,16], but bi- or multimodal peaks are also sometimes observed [15,30].

More recently, a time-frequency analysis approach has been developed by which the time-frequency distribution is decomposed into a number of descriptive functions reflecting second-to-second variations in fundamental frequency and waveform morphology [31,32]. The main advantage of using this approach is that the local signal quality and structure is also assessed. The spectral profile differs from the conventional spectrum in that the different local spectra have been frequency aligned before averaging. This results in more distinct peaks and a more clearly discernable harmonic structure. With its second-to-second resolution, the method can rapidly detect changes in frequency, regularity, amplitude, waveform and signal structure and quality (Figure 1).

To date, the dominant spectral peak has been presented as either dominating atrial cycle length (in ms), fibrillatory rate (in fpm) or fibrillatory frequency (in Hz). Since it has become evident that the results of this method might be of practical clinical importance, we believe that the results should be expressed in a way which is closest to the nomenclature of other surface ECG rate-variables (e.g. sinus rate, atrial tachycardia or flutter rate) [33]. The expression “fibrillatory rate”, with its unit “ibrillations per minute - fpm” may therefore be most appropriate [20]. Moreover, the calculation of cycle length from the frequency power spectrum with its standard unit Hz (cycle length in ms = 1000/frequency in Hz) seems somewhat troublesome [34]. While this calculation is appropriate for single measurements, its application to multiple simultaneous or repeated measurements such as pre- and post-drug states may introduce significant statistical and subsequently scientific errors, especially if the baseline frequency exhibits large inter-individual variability. The reason is, that the same frequency difference results in larger cycle length differences for low frequencies compared to high frequencies.

### Method Validation and Reproducibility

**Figure 2:**
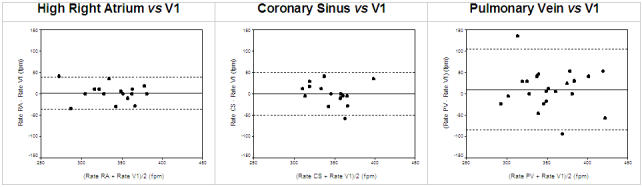
Agreement of fibrillatory rates from the right atrium (upper left), coronary sinus (upper right), superior pulmonary veins (lower) with simultaneously recorded fibrillatory rates from surface ECG lead V1 (Bland-Altman method).

A direct comparison between endocardially recorded electrograms and body surface recordings clearly evidences the validity of fibrillatory rate obtained from surface ECG as an index of the average atrial fibrillatory cycle and subsequently atrial refractoriness. Fibrillatory rates calculated from lead V1 substitute the right atrial free wall [15,16] whilst rates from an oesophageal lead reflect atrial septal and left atrial activity [15]. Figure 2 illustrates the close agreement of right atrial and coronary sinus fibrillatory rates with rates obtained from simultaneously recorded surface ECG lead V1 in persistent AF. Interestingly, rate differences and variability between ECG and electrograms increase with growing anatomical distance (right atrium - coronary sinus - pulmonary veins) to V1. Approximately 50 % of V1 and superior pulmonary vein rates are also within close range. In the other cases, the latter has been found to be substantially faster but also slower than V1 (right atrium), indicating that superior pulmonary veins might not necessarily be the driving source of the fibrillatory process.

Although the gross atrial fibrillatory pattern, studied by multiple simultaneous epicardial recordings, is reproducible already in repeated measurements of only 8 seconds duration [35], calculation of fibrillatory rates during steady state conditions from even longer time intervals discloses a true variability. Thus, increasingly longer recording times of up to 30 minutes result in a successive decrease of this variability. The reproducibility of the method is thus enhanced by prolonging the recording time during steady state conditions, allowing integration of atrial fibrillatory activity over longer periods. For practical reasons, steady state recordings may be restricted to one to five minutes, yielding rate variation coefficients of 2.1 % [15].

In persistent AF, there is minor short-term rate variability [15,16,27] and considerable diurnal variability (for details see below) [23] [24], whilst repeated daily frequency measurements at identical medication at the same time under similar conditions discloses an insignificant fibrillatory rate variability [36]. In contrast, rate variability in paroxysmal AF seems to be related to its natural course with a rate increase within the first five minutes of an AF episode [22] and a rate decrease prior to termination [22,28].

## Exploring Autonomic Modulation of AF

Vagal as well as sympathetic stimulation have been shown to reduce atrial refractory periods and increase their heterogeneity [37-39]. In the human atrium beta stimulation has been found to predominate over vagal stimulation [38]. Moreover, invasive studies performed in subjects with sinus rhythm have suggested a circadian pattern in atrial refractoriness with longer refractory periods during night time and refractory period shortening during daytime [40,41] which supports the role of the autonomic nervous system in modulating atrial electrophysiologic properties.

Subsequently, atrial fibrillatory rate seems to be ideal for monitoring the effect of autonomic tone changes - either spontanenous (circadian) or by autonomic maneuvers provoked - on atrial electrophysiology. Indeed, the circadian variability of atrial fibrillatory rate has been explored in independent studies [23,24]. Fibrillatory rate obtained from Holter ECG’s with persistent AF showed a significant decrease at night and an increase in the morning hours studies [23,24]. In 6 out of 30 individuals studied by our group studies [23], dominant nocturnal fibrillatory rate increased, however, concomitantly with a decrease in ventricular rate, while the opposite change occurred in the morning hours.

The second area of investigation concerns effects on atrial fibrillatory rate following vagal or sympathetic stimulation during experimental conditions. Carotid sinus massage, supposed to mainly induce vagal stimulation, resulted in a variable effect on fibrillatory rate in 19 patients studies [42]. A reproducible decrease was noted in 9 individuals, whilst a rate increase occurred in 8 and no change was observed in two patients. Interestingly, calcium-channel blocker treatment was the only variable effecting the rate response to carotid sinus massage. Calcium-channel blockers were more frequently used in patients with a decrease in fibrillatory rate compared to patients with a rate increase. The effect of adrenergic stimulation via head-up tilting on fibrillatory rate was studied in 14 patients with long-lasting AF studies [43]. In 12 patients head-up tilting increased fibrillory rate significantly, while there was no rate change in the remaining two.

With the availability of instantaneous fibrillatory rates obtained from time-frequency analysis, their second-to-second variation can be explored by spectral analysis techniques, similar to those used for analyzing heart rate variability. By applying this approach, Stridh et al studies [44] noted a spectral peak in 2 out of 8 patients with permanent AF at the breathing frequency of 0.125 Hz during controlled respiration that disappeared after atropine injection.

All these findings together clearly highlight the complexity of autonomous nervous system effects on atrial electrophysiology and justify further exploration.

## Monitoring and Predicting Antiarrhythmic Drug Effects

Class I and III antiarrhythmic drugs have been shown to increase atrial cycle length (decrease fibrillatory rate) which coincides with increased refractoriness and decreased conduction velocity [45]. These mixed effects might explain why atrial cycle length is closely related with baseline refractoriness [6] but not with refractoriness after antiarrhythmic drug administration [46]. Even though the individual contribution of refractoriness prolongation or conduction slowing might currently not be differentiated, monitoring drug action on atrial fibrillatory rate seems well fit to be explored by analysing the surface ECG. Indeed, a substantial reduction in atrial fibrillatory rates following several different intravenously or orally administered class I and III antiarrhythmic drugs as well as following verapamil or magnesium  has been observed (Table 1).

**Figure 3:**
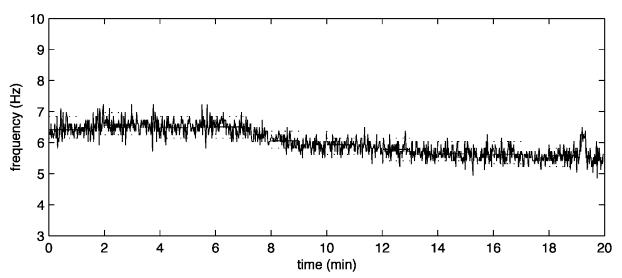

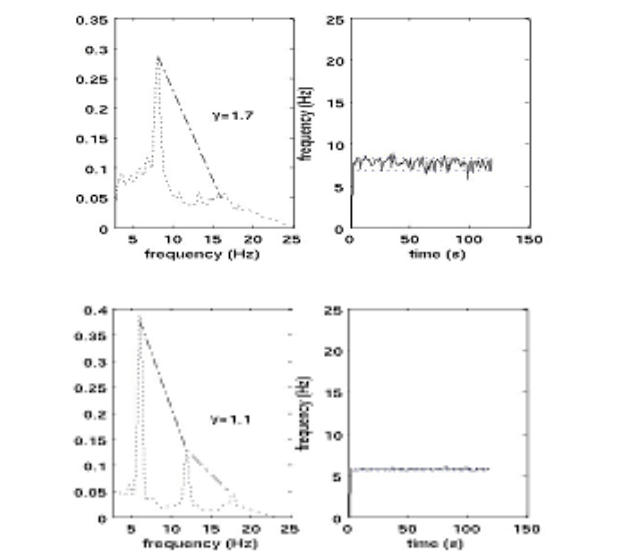
Examples of antiarrhythmic drug monitoring. Atrial fibrillatory rate obtained from time-frequency-analysis during **intravenous infusion of dl-sotalol**. The solid black colour indicates the actual fibrillatory rate. Initially, the atrial rate is at the level of 390 fpm (6.5 Hz), but decreases successively to the level of 330 fpm (5.5 Hz) during 20 minutes (upper panel). Time-Frequency-analysis at baseline (middle) and after 3-days **oral flecainide** intake (bottom). Frequency power spectrum (left) and frequency trend (right). Please note the more pronounced dominant and harmonic peaks after drug administration as well as atrial rate reduction and rate stabilization.

Two examples of drug monitoring are presented in Figure 3 showing the transition from a high rate (less organized) to a low rate (more organized) fibrillation following acute intravenous sotalol infusion or 3-day oral flecainide administration.

**Figure 4:**
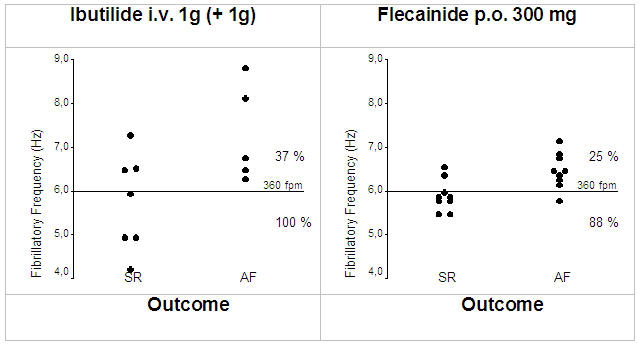
Relation between baseline fibrillatory rate and response to intravenous ibutilide (left) [16] and oral flecainide (right) [19]. AF with slower rates is more likely to respond to antiarrhythmic drug therapy, while faster rates are more often found in drug-refractory AF.

Besides direct monitoring of antiarrhythmic drug effects, it seems also possible to identify suitable patients for pharmacological cardioversion. A baseline fibrillatory rate of 360 fpm was highly sensitive and specific for prediction of AF termination following intravenous ibutilde [16] or oral flecainide [19] (Figure 4). In contrast, Fujiki et al noted no baseline fibrillatory rate difference between patients who converted to sinus rhythm and those who did not following oral bepridil administration. Instead, larger rate increases were associated with AF termination. While this concept is interesting and well established in experimental AF [45], it needs to be emphasized that these authors calculated atrial cycle length from the frequency spectrum, which may have introduced the aforementioned statistical errors [34].

Patients with a low fibrillatory rate may have a small number of wavelets (long wavelength), whereas those with higher rates have multiple wavelets (short wavelength) [7]. In the former group class I or III antiarrhythmic drugs by decreasing fibrillatory rate may have increased wavelength (or the excitable gap) and therefore reduced the number of wavelets that could coexist. This would have increased the statistical chance that all wavelets might extinguish simultaneously and terminate the fibrillatory process [47].

One previous study suggested that a stepped conversion regimen of first-line ibutilide followed by electrical cardioversion for patients who fail to convert is less expensive and has a higher conversion rate than first-line electrical cardioversion [48]. Given the expense of antiarrhythmic therapy and the risk of side effects including ventricular proarrhythmia, a test that differentiates responders from non-responders is likely to be even more cost-effective. In addition, drug monitoring using frequency analysis techniques may be useful for finding optimal drug dosages and timing of interventions in the individual patient.

## Predicting AF Recurrence

Previous investigations [49,50] have shown that most AF relapses occur within the first weeks after cardioversion with decreased but constant recurrence rates thereafter. Early vulnerability to AF re-initiation within this time period is related to electrophysiological abnormalities, while structural abnormalities seem to be primarily responsible for later AF recurrences [51]. This time course might be explained by the fact that reversal of the electrical remodeling process occurs rapidly once sinus rhythm is restored [12,52,53], while structural changes persist for longer periods [51]. Subsequently, characterization of atrial electrophysiology has been suggested for identification of patients at risk for early AF recurrence [2].

Atrial premature beats with short coupling intervals have been shown to promote early AF reinitiation following cardioversion [54,55]. AF reinduction by an atrial premature beat relies on the fact that a relatively short atrial wavelength (conduction velocity x refractory period) must be present [56]. As highlighted before, atrial fibrillatory rate reflects atrial refractoriness [5,6], and might subsequently represent a marker for early AF susceptibility following restoration of sinus rhythm. Indeed, previous studies reported higher rates in relapsed patients immediately prior internal [57] or external cardioversion [21] when compared with non-relapsed patients and also close relationships between fibrillatory rate and defibrillation thresholds [57,58].

**Figure 5:**
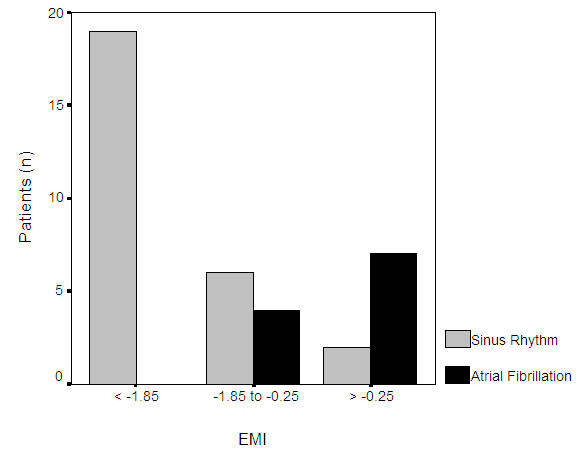
Outcome following cardioversion stratified by an “electromechanical index” (EMI) according to the regression equation (EMI = 0.176 systolic left atrial area + 0.029 fibrillatory rate - 17.674) [21]

Two previous studies [21,59] have investigated the combined predictive value of fibrillatory rate and echocardiographic left atrial parameters. In one study [59], the authors calculated an index combining shortest cycle length from oesophageal or V1 lead and standard left atrial diameter and were able to show that patients with recurring AF had significantly lower values than patients who remained in sinus rhythm. Another study [21] demonstrated that the combination of fibrillatory rate and systolic left atrial area predicted early AF recurrence after succesful cardioversion with a high accuracy and was able to provide individual risk estimates (Figure 5).

These parameters seem therefore well suited to describe the individual atrial remodeling. The concept of ECG-guided cardioversion may gain even greater importance in the light of previous findings from two studies comparing rate- with rhythm-control strategies [60,61]. In these studies, rhythm-control seemed not to be superior than rate-control in asymptomatic, mostly eldery patients with recurring AF and structural heart disease. This highlights the need to select candidates for cardioversion not only based on clinical judgement but also on measures that are able to determine the likelihood of maintaining sinus rhythm such as rate parameters obtained from the surface ECG.

## Conclusions

Atrial fibrillatory rate and its variability can be reliable obtained from the surface ECG in AF patients using spectral analysis techniques. These parameters exhibit a significant interindividual variability allowing individual quantification of the atrial electrical remodeling process. Frequency analysis of AF might prove useful in identification of underlying AF pathomechanisms, and prediction of therapy efficacy (drug-induced conversion, maintenance of sinus rhythm, selecting antiarrhythmic drugs, identifying candidates for non-pharmacological AF therapy). Further larger studies are necessary to determine the role of these techniques in different AF management strategies in order to select and time the appropriate therapy for the individual patient.

## Figures and Tables

**Table 1:**
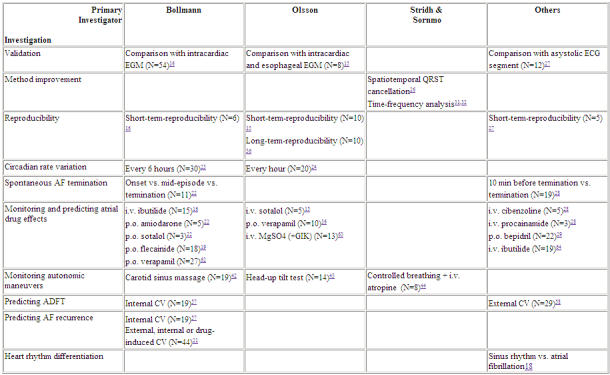
Summary of available literature on frequency analysis techniques (for details see text)

CV = cardioversion, EGM = electrogram, GIK = glucose, insulin, potassium, ADFT = atrial defibrillation threshold
